# The sound of science: Data sonification has emerged as possible alternative to data visualization

**DOI:** 10.1038/s44319-024-00230-6

**Published:** 2024-08-19

**Authors:** Lutz Bornmann

**Affiliations:** Science Policy and Strategy Department, Administrative Headquarters of the Max Planck Society, Hofgartenstr. 8, 80539 Munich, Germany

**Keywords:** History & Philosophy of Science, Science Policy & Publishing

## Abstract

Sonification uses sound to display larger datasets as an alternative to graphic displays. While it has some advantages over visualization, sonification is still largely limited to a few specialized applications and public outreach projects.

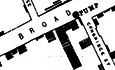

Following the wisdom that “a picture says more than a thousand words”, scientists have been using visualization of data extensively to display, interpret, and present their results. Graphical representation of data has been integral to research and communication: Examples range from John Snow’s 1854 hand-drawn map of Soho, London (Fig. 1) that the British physician used to track the source of a cholera outbreak to the interactive “landscape of biomedical research” (https://static.nomic.ai/pubmed.html) that displays more than 33 million scientific papers from the past 50 years. Data visualization has become so dominant and important for science that it is now a research field in its own right, including funding schemes, journals, and regular conferences. Dayé and de Campo (2006) explain this predominance of visual representation by the fact that Western culture is largely visually oriented: “It is a culture of seeing, of reading, a culture of scripture and images. Science, as a part of this culture, has relied and continues to rely on the perceptual capacities of the eye.”

**Figure 1:**
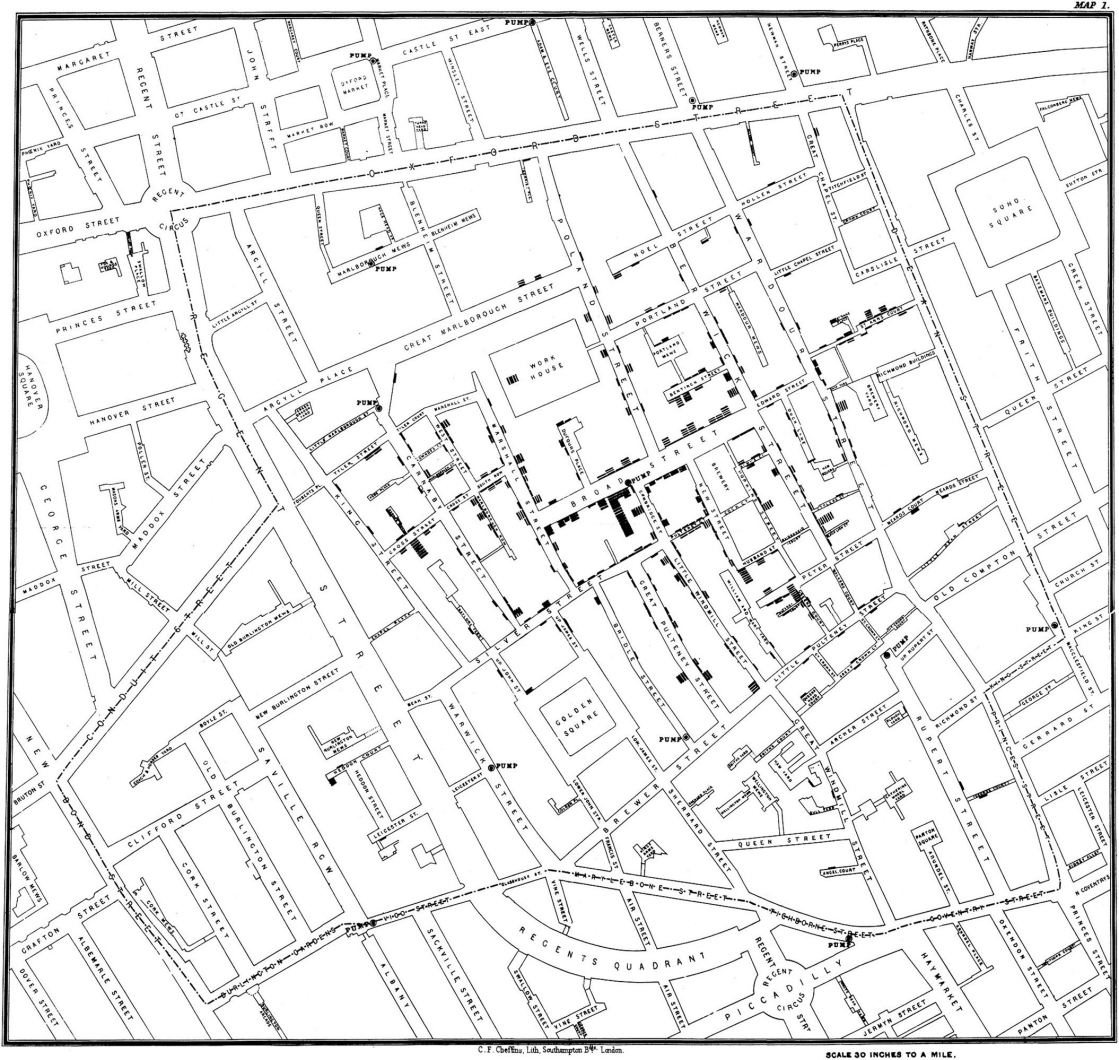
Original map made by John Snow in 1854 that shows the clusters of cholera cases (indicated by the black rectangles) in the London epidemic of 1854. The map is one of the first examples of data visualization to better understand the pattern of cases and prove Snow’s theory that cholera spread through water systems. The contaminated pump is at the intersection of Broad Street and Cambridge Street. Originally published in Snow (1855). Wikipedia/Public Domain.

However, humans possess more than one sense and there is no biological reason not to use the other ones. Visually impaired people, for example, compensate with a more acute sense of hearing, smell or feeling. Hence, scientists, engineers as well artists and others have explored ways to use other senses than just the eyes to transmit and analyze information. The next obvious one would be hearing as humans not only evolved a highly sensitive and capable sensing organ for hearing but also the brain circuitry to quickly process large amounts of audio information. As a result, the field of data sonification has emerged during the past decades as a viable alternative to represent and analyze scientific data; yet it still remains focused on specialized applications rather than a viable alternative method to visualization.

“…humans not only evolved a highly sensitive and capable sensing organ for hearing but also the brain circuitry to quickly process large amounts of audio information.”

Sonification involves translating data into sound for presentation. Probably one of the first known examples is Galileo Galilei’s experiment using a ball rolling down an inclined plane: “A small heavy ball was released down an inclined plane so that as it rolled it lightly touched catgut strings that were tightened above the plane […] Galileo noticed that every time he repeated the experiment, the sound of the strings had the same rhythm” (Dayé and de Campo, 2006). Modern examples of data sonification include the Geiger counter, which “displays” ionizing radiation detection with crackling noises, the pulse oximeter, a medical device that emits a tone correlating with a patient’s blood oxygen level, or the heart rate monitor that sonifies a patient’s heartbeat, both aiding clinicians during surgery.

The emergence of a specialized community that uses sound to reflect properties of data began in the late 1980s with the advent of microprocessors capable of generating sound from digital data. Scientists started research projects on augmenting graphical user interfaces with audio and utilizing sound for the analysis of datasets. The International Conference on Auditory Display (ICAD) was first convened by Gregory Kramer in 1992 at the Santa Fe Institute in the USA, marking the beginning of a dedicated research community (Supper, 2015). In recent years, data sonification has been further developed as a complement or an alternative to data visualization in isolated research projects. The projects remain largely limited to specialized applications, public outreach and communication of research. Lindborg et al (2023) describe data sonification as “an emerging discipline that struggles to define its boundaries, its impacts and more importantly, shared methods, processes, and tools for the mapping of data to sound.”

“In recent years, data sonification has been further developed as a complement or an alternative to data visualization…”

## Definition of sonification and its history

Data sonification links sound elements to data points, similar to how visualizations links graphical elements to data. It falls under the category of auditory displays, defined as displays utilizing sound to relay information (Walker and Nees, 2011). For Kramer et al (2010), “sonification is defined as the use of nonspeech audio to convey information. More specifically, sonification is the transformation of data relations into perceived relations in an acoustic signal for the purposes of facilitating communication or interpretation.” An expanded definition by Liew and Lindborg (2020) describes sonification as “any technique that translates data into nonspeech sound, with a systematic, describable, and reproducible method, in order to reveal or facilitate communication, interpretation, or discovery of meaning that is latent in the data, having a practical, artistic, or scientific purpose.”

While Kramer et al (2010) and Liew and Lindborg (2020) specifically exclude spoken audio in their definitions of sonification, other researchers take a more liberal view: “it is hard to uphold that ‘visualization’ should exclude visual symbols such as text, numbers, emojis etc., at least in practice” (Lindborg et al, 2023). In scientific literature, it is norm to detail figures in manuscripts to make these self-explanatory and spoken audio can serve a similar clarifying role in sonification: “it is often hard to understand quantities from sonifications, but speech provides the user with exact quantifiable information” (Nasir and Roberts, 2007). Unlike visualizations, which are typically integrated into scientific papers, sonifications often exist as standalone outputs. It is therefore helpful for listeners if sonifications are explained in more detail. For example, Bornmann and Haegner (2024) used spoken audio not only to explain their specific sonification of bibliometric data but also to appreciate the researcher to whom the sonification is dedicated.

Data sonification is a multidisciplinary field, incorporating elements from human perception, acoustics, design, arts, and engineering that brings together professionals with diverse backgrounds, including psychologists, computer scientists, engineers, physicists, composers, and musicians. Other disciplines integral to data sonification include audio engineering, audiology, informatics, linguistics, mathematics, psychology, and telecommunications (Walker and Nees, 2011). Data sonification has been employed in various projects to analyze data, monitor complex systems, aid in data navigation or to identify patterns and structures. Walker and Nees (2011) outline three primary functions of data sonification: alerts, alarms, and warnings, where sounds prompt immediate action; status and monitoring messages that reflect the current state of a system; and data exploration by encoding and handling dataset information into sound.

“Data sonification is a multidisciplinary field, incorporating elements from human perception, acoustics, design, arts, and engineering…”

Among the various methods for data sonification, parameter mapping has emerged as the predominant contemporary technique. This is not a simple conversion of data into sound but various parameters of the data are represented by manifold sound events, such as loudness, pitch, timbre or duration (Dayé and de Campo, 2006). The input (data) and the output (sound) are linked through a complex mapping schema that delineates their relationship (Lindborg et al, 2023).

## Advantages and drawbacks

Data sonification has specific benefits and some advantages over visual representation of data. First, the human auditory system is highly adept at discerning temporal patterns and complex sound properties, which makes it a powerful tool for processing information. The ear is also one of the most precise sensory organs of the human body able to distinguish sound frequencies with a high resolution. Auditory displays “exploit the superior ability of the human auditory system to recognize temporal changes and patterns […] As a result, auditory displays may be the most appropriate modality when the information being displayed has complex patterns, changes in time, includes warnings, or calls for immediate action” (Walker and Nees, 2011). As Hermann et al (2011) put it, untrained listeners may notice “that ‘something is wrong’ with their car engine, just from its sound, whereas a professional car mechanic can draw quite precise information about the detailed error source from the same sound cue.”

Second, sonification offers a broader range of design possibilities than visualization, as sound is characterized by various parameters, such as pitch, volume, tempo, location in a stereo field or timbre (Zanella et al, 2022). Moreover, many people can still hear and efficiently process information from audio while they are busy with other tasks. In fact, podcasts that explain and explore specific topics—while obviously dominated by spoken language—have become popular specifically because people can listen to them while doing other, mundane tasks such as driving or performing boring, repetitive jobs in the lab.

Third, sonification enables access to scientific knowledge for people who are visually impaired. Noel-Storr and Willebrands (2022) interviewed four blind or visually impaired astronomers about the specific challenges to access data. Garry Foran from the Centre for Astrophysics and Supercomputing at Swinburne University of Technology carries out his research work by collaborating with sonification tools developers “that facilitate the management and analysis of astrophysical data using sound”. For Nic Bonne from the Institute of Cosmology and Gravitation at the University of Portsmouth, it is challenging that “a lot of astronomers see sonification as a gimmick and not something with practical applications. There is work to be done to make sonification more mainstream and show that it is a valid way of analyzing data.”

Fourth, in practical work settings, such as an operating theater, workers frequently find themselves unable to view or see a visual display. Environments in which “large numbers of changing variables and/or temporally complex information must be monitored simultaneously are well suited for auditory displays” (Kramer et al, 2010). Our hearing is constantly active, making it valuable for monitoring alarms and continuous data streams (Zanella et al, 2022). Combining sound with visualization enhances the effectiveness of data overview and feature identification.

“Environments in which “large numbers of changing variables and/or temporally complex information must be monitored simultaneously are well suited for auditory displays.”

Fifth, sonification adds an element of enjoyment to the utility of data exploration, akin to listening to music.

However, the specific benefits of data sonification come with drawbacks. Most individuals are not as skilled in recognizing auditory patterns as they are with visual patterns, necessitating additional training to fully utilize sonification as a tool (Kramer et al, 2010). Furthermore, there is a risk that sonification may not aid in data interpretation. “By focusing mostly on technical questions about how data can be made audible, the interpretation of the underlying data often slips into the background” (Supper, 2015). Involving empirical domain experts in the sonification process can mitigate this risk. Lastly, sound-based decisions in sonification may seem subjective and arbitrary, potentially making them unsuitable for data analysis. Establishing widely accepted standards could address the subjectivity and uncertainty in sonification, although this is complicated by cultural and social aspects in questions of sound and music.

## Between data and music

Since its start in the late 1980s, sonification has been applied across various fields such as seismology, astronomy, geography, cartography, and the social sciences. While some projects adopt sparse musical styles, others opt for more abstract soundscapes, blurring the lines between science and art: “Although some sonifications are couched in a relatively traditional musical idiom, such as the jazz of the Microbial Bebop project, many eschew traditional musical conventions in favor of more abstract sounds. The applications for which sonifications are developed range from analysis tools for scientific specialists to musical pieces; in fact, many projects playfully straddle the boundary between science and art” (Supper, 2015).

“The applications for which sonifications are developed range from analysis tools for scientific specialists to musical pieces; in fact, many projects playfully straddle the boundary between science and art”.

While data sonification incorporates musical elements through parameter mapping, its classification as music is debatable. The Data Sonification Archive (https://sonification.design) contains many sonifications that may not qualify as musical, aesthetic or emotional experiences. The central goal of sonification is the representation of data: “Music and sonification have ostensibly different goals. The composer strives for aesthetic interest […] In sonification, it is not aesthetic interest but the successful signification of the data that is the goal” (Vickers, 2016). Despite differing objectives, there is a trend of blending music with data sonification and sonification concerts have been integral to ICAD meetings. The inclusion of musical elements in sonifications is often justified by the need to maintain listener engagement: “a sonification that is more musical will be better than one that is not” (Vickers, 2016). However, Walker and Nees (2011) emphasize that sonifications should not lose their intended message amidst musical considerations and advocate to “design aesthetically pleasing sonifications to the extent possible while still conveying the intended message”, prioritizing information exchange over musicality.

## Data sonification for public outreach

Incorporating musical elements into sonification of scientific data creates tracks that may be effective in reaching out to non-scientific audiences, especially younger people. Disseminating research findings to the public is essential. The current post-academic era expects such knowledge transfer from the scientific community through traditional media or social media. Larsen and Gilbert (2013) emphasize the importance of knowledge transfer for informed policy decisions on complex issues like climate change: “In order for society to make effective policy decisions on complex and far-reaching subjects, such as appropriate responses to global climate change, scientists must effectively communicate complex results to the non-scientifically specialized public.” The authors propose sonification as an effective tool in this respect. In the following, two projects are described that use data sonification to specifically reach out to non-scientific audiences.

The sonification project by Larsen and Gilbert (2013) was inspired by bebop jazz to create “aesthetic musical compositions that interpret the relationships between elements in large biological datasets.” For the conversion, they developed an algorithm for translating vast amounts of data into music that sounds similar to contemporary jazz. Termed “microbial bebop”, their musical representation is crafted from a five-year sequence of oceanic microbial activity and environmental parameters, including temperature, salinity, and chlorophyll levels (https://soundcloud.com/plos-one-media/sets/microbial-bebop). The sonified dataset forms a fraction of the continuous scientific records maintained at the Western Channel Observatory (https://www.westernchannelobservatory.org.uk) since the early 20th century. Peter Larsen explained the rational to sonify the data as follows: “It’s my job to take complex datasets and find ways to represent that data in a way that makes the patterns accessible to human observations. There’s no way to look at 10,000 rows and hundreds of columns and intuit what’s going on” (Madhusoodanan, 2013). He especially emphasizes the outreach function of data sonification: “an added benefit of turning […] data into music is its ability to reach people who gravitate more toward art than science” (Moskowitz, 2012).

The second project has been published by Miriam Quick and Duncan Geere on Loud Numbers (https://loudnumbers.bandcamp.com/track/the-end-of-the-road). “The End of the Road” serves as an elegy to the dwindling diversity of species by sonifying the decline of insect populations. The data for the project was derived from a study by Møller (2019) who recorded insect fatalities on his car’s windscreen over a span of twenty years, while driving on two specific routes in Denmark with near-daily regularity each summer. The findings of his study revealed a staggering 80 to 97 percentage points decrease in insect numbers from 1997 to 2017.

The sonification features two distinct data-driven elements. The monthly tally of insects that met their end on the author’s windshield is audibly translated into a series of fluttering synthesizer tones. The pitch of these tones is indicative of the insect size, with higher pitches denoting smaller insects and lower pitches for larger insects. A noticeable reduction in insect counts leads to a corresponding diminishment in sound, resulting in an increasingly barren auditory landscape. A synthesizer pad echoes a descending melody, mirroring the global terrestrial insect population’s annual decline of 1.1 percentage points. With every 5 percentage points decrease in insect numbers, the melody descends by one note.

The data sonification by Miriam Quick and Duncan Geere encapsulates the experience of a solitary drive through an expansive, empty terrain, punctuated by the sound of insects colliding with the windshield. Ambient sounds of the environment, such as the whoosh of passing cars and the melodies of bird calls, add to the soundscape. The texture of the track grows more minimalistic as it progresses, reflecting the vanishing insect life. A somber bell toll marks each year documented within the data, as a requiem for biodioversity loss.

Another example includes the track “Talkative Men—The Gender Difference in the Zurich Cantonal Council” (https://github.com/simonhuwiler/nzz_zh_kantonsrat_sonofication) from the social sciences. The track is based on survey data and sonifies how men and women contribute to a debate of the Zurich Cantonal Council in Switzerland. A recent example is the “Bristol Burning” project from Miriam Quick, who turned air quality data into a track and combined audio with video elements (https://youtu.be/AhWWO0EtM1c). The track can not only be used to explore air quality data but her cooperation with the hip-hop artist T. Relly also raises it to a musical delight. Data sonification projects in the area of climate change are documented by Lindborg et al (2023). Many projects have a public outreach background. A summarizing study by Zanella et al (2022) shows for astronomy that more than 60% of sonification projects combine sound with visual elements to foster public engagement in research.

## Discussion

Visualizations are the dominant method for presenting empirical findings in both scientific and broader contexts. Dayé and de Campo (2006) suggest that sonification is not meant to replace visualization but rather to complement it. They advocate for a holistic approach that utilizes the strengths of all senses: “the thoughtful use of all the human senses, making good use of the perceptual strengths of each, is a more complex, but ultimately more fruitful undertaking. Where possible, it is advisable to combine both modes of perceptual representation, vision and hearing.”

“Where possible, it is advisable to combine both modes of perceptual representation, vision and hearing.”

As visualizations play such a dominant role in scientific dissemination, only few can interpret sonifications effectively. “We learn how to read graphical displays, and scientists’ skills are often highly developed and sophisticated in this area. But we do not learn how to identify structures or patterns in a given sequence of sounds” (Dayé and de Campo, 2006). To enhance understanding, sonifications may require additional training or the inclusion of visual and explanatory elements, such as spoken audio.

The intersection of science, art, and public outreach through data sonification offers significant benefits. While scientific communication typically involves text, numbers, and formulas, artists engage with a diverse array of mediums including movies, images, sound, and sculpture. Agné et al (2020) recognize the synergy between arts and sciences as a catalyst for intuitive and creative thinking, which can also foster public literacy and engagement with societal issues. The multi-sensory communication of empirical data enables “users to hear, see, explore, and intuitively understand large amounts of information in as short a period of time, and with the minimum cognitive load possible” (Worrall, 2019).

To explore the potential benefits of data sonification over visualization, more and dedicated research is essential to analyze the best ways to display and comprehend empirical data (Dayé and de Campo, 2006). Experiments should be conducted to verify if sonifications achieve their intended goals, such as helping listeners to understand specific data structures (Worrall, 2019). With more evidence that sonification could be as good as visualization—at least for specific data types or applications —along with training to better discern patterns and structures from sound, sonification may indeed become a viable alternative to the prevalent figures and illustrations in scientific presentations and publications.

## Supplementary information

Peer Review File
