# Supplementary material for: The sound of science: Data sonification has emerged as possible alternative to data visualization
Source: EMBO Rep. 2024 Aug 19;25(9):3743–7. doi: 10.1038/s44319-024-00230-6 (PMC11387736; doi:10.1038/s44319-024-00230-6)
Supplement: Supplementary file 1 — Peer Review File [file 44319_2024_230_MOESM1_ESM.pdf]

EMBO reports

The sound of science. Data sonification has emerged as possible alternative to data visualization

Lutz Bornmann

Corresponding author(s): Lutz Bornmann (bornmann@gv.mpg.de)

Review Timeline:

Submission Date:	8th Aug 24
Accepted:	9th Aug 24

Editor: Holger Breithaupt

Transaction Report: This article was editorially reviewed.
